# Clinical and morphological pattern of brain arteriovenous malformations (BAVMs) in a Tertiary Care Hospital in Bangladesh

**DOI:** 10.1186/s13104-015-1717-4

**Published:** 2015-12-05

**Authors:** Ahmed Hossain Chowdhury, Sharif Uddin Khan, Kazi Mohibur Rahman, A. T. M. Hasibul Hasan, Swapon Kumar Ghose, Badrul Haque, Mansur Habib, Quazi Deen Mohammad

**Affiliations:** Department of Neurology, Dhaka Medical College Hospital-2, 3rd Floor, Ramna Dhaka, Bangladesh; National Institute of Neurosciences and Hospital, Dhaka, Bangladesh

**Keywords:** Arteriovenous malformation (AVM), Intracranial hemorrhage, Feeding vessels

## Abstract

**Background:**

We have conducted this study to examine the clinical and morphological pattern of brain arteriovenous malformations (BAVMs) along with their treatment and short term outcome in a tertiary care hospital in Bangladesh. This retrospective chart review was carried out from the records of neuro-endovascular division at Department of Neurology, Dhaka Medical College Hospital (DMCH) from January 2010 to June 2013. A total 60 patients were evaluated. All the necessary information regarding the demographic, clinical, morphologic and treatment profile was gathered through a predesigned questionnaire. To our knowledge, we have the largest cohort of BAVM patients in Bangladesh and this is the first of this kind of study done in Bangladesh.

**Results:**

The mean age at diagnosis was 30.3 years with a standard deviation of ±14.3 and the majority was teenagers (30 %). Intracerebral hemorrhage was the commonest (70 %) type of presentation at diagnosis, followed by headache (50 %), altered consciousness (50 %), vomiting (40 %) and seizure (40 %). Majority of the AVMs had feeders from anterior circulation (50 %) and most of the AVMs (73.3 %) were supplied from the main feeders, whereas the rest from distal vessels. Regarding venous drainage, AVMs drained mostly either to superficial (43.3 %) or deep (40 %) venous system. AVMs frequently had larger (40 %) nidus size and a slow to medium flow (60 %), through the nidus. An eloquent AVM location was found in 50 % of the patients. Intranidal aneurysm was found in 10 % AVM and angiopathic AVM in 13.3 %. Patients were treated by endovascular embolization (31) or surgical excision (11) or conservative approach. There was one event of death, both in embolization group and surgically treated group before discharge. The patients were followed up for 1.3 ± 0.8 years. The rate of rebleed was 6.6, 30 and 60 % during follow up in endovascular, surgical and conservatively treated group. Though five patients in conservative group died during this time, no deaths reported in intervention group (endovascular or surgery).

**Conclusion:**

Intracerebral hemorrhages, headache, altered consciousness and seizure are common clinical presentations of AVM at diagnosis. The remarkable morphologic features are larger AVM size at eloquent location, medium to slow flow with frequent feeders from main vessels of anterior circulation and drainage to superficial venous systems. Endovascular embolization or surgical excision of AVM are relatively safe and effective and provides better short term outcome than conservative approach.

## Background

Brain AVMs are known since the middle of 19th century [1, 2]. The first clinical description of brain AVMs was published by Steinheil [3] and Hoffman [4]. With the introduction of cerebral angiography by Moniz [5] the awareness regarding this relatively unknown condition also started to increase among the physicians. It has long been thought to arise from developmental disruption of vessel formation either at embryonic [6] and the fetal stage [7], or after birth [8]. Their course may be rather unpredictable; they may remain static, grow, or even regress [9]. Brain AVMs come to clinical attention mainly in young adults, typically before the age of 40 [10]. The prevalence of BAVMs is difficult to estimate, given the lack of a population subject to uniform brain imaging. Some insights are provided from rare studies suggesting the prevalence up to 300,000 (0.1 %) persons in the United States [10, 11]. In the general population the estimated rate is approximately 0.01 %, but reported rates range from 0.001 to 0.52 % [11–15]. Hospital based post mortem series have reported brain AVM prevalence of up to about 600 per 100,000 [16–18]. As yet, there has been lack of population based studies specifically investigating brain AVM prevalence [19].

The goal of AVM treatment is mostly prevention of hemorrhage which is achieved by microsurgery, embolization or radio surgery [20]. Let alone the epidemiologic studies, we did not even had any hospital based study on brain AVMs in Bangladesh. We knew very little regarding the angioarchitecture of brain AVMs before the introduction of diagnostic digital subtraction angiography (DSA) facilities in Bangladesh. DSA evaluation of such disorders started in 2006 at Dhaka Medical College Hospital (DMCH). Though the clinical and morphological features of BAVM are not unknown, we conducted this study as because there was no such study in Bangladesh done previously and we had the largest cohort of AVM patients in our center. We wanted to delineate the clinical picture along with the morphologic features at angiography and short term treatment outcome of these patients in Bangladesh.

## Methods

This is a retrospective chart review. We reviewed the records from Neuro-endovascular division at the Department of Neurology, DMCH from January 2010 to June 2013.

DMCH is the one of the highest center for referral of all type of medical problems in Bangladesh. It is a 2300 bed hospital in the heart of the capital and receives patients from all over the country. The Department of Neurology also provides inpatient care through 36 hospital beds in addition to its regular outpatient services. The Neuro-endovascular division is headed by an Associate Professor of interventional neurology who is trained in neuro-intervention at a renowned center outside Bangladesh.

**Fig. 1:**
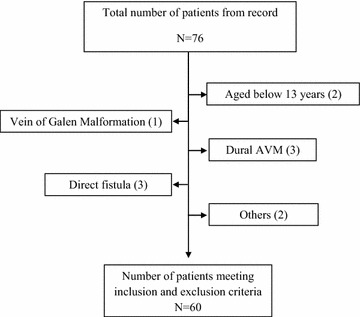
Flow chart of patients (number) included and excluded for each cause

Study population initially included 76 patients from the hospital records over this period of time. Later on, 60 patients were selected finally, following the inclusion and exclusion criteria (Fig. 1). Information regarding the demographic, clinical and morphological profile was gathered through a predesigned questionnaire. Demographic characteristics assessed were age at date of diagnosis and sex. Clinical characteristics at presentation assessed were intracranial hemorrhage (parenchymatous, intraventricular, or subarachnoid), generalized or focal seizures, chronic headache, altered consciousness, vomiting, vertigo, sense of imbalance, visual or speech disturbance and reversible, persistent, or progressive neurological deficits. Morphological characteristics assessed were classified in all databases according to the Spetzler–Martin grading system [21] with its three element sizes (small, maximum diameter, 3 cm; medium, diameter 3–6 cm; and large, diameter 6 cm), drainage (any venous drainage component into the deep, i.e., internal cerebral veins), and location in so-called “eloquent” brain regions (“the sensorimotor, language, and visual cortex; the hypothalamus and thalamus; the internal capsule; the brain stem; the cerebellar peduncles; and the deep cerebellar nuclei”).

## Inclusion criteria

- Patients aged 13 years and above.
- Diagnosed and confirmed as a case of Brain AVM previously with the help of magnetic resonance arteriography or digital subtraction angiography.
- Consensus about the diagnosis among at least two interventional neurologists of the center.

## Exclusion criteria

- Patients aged below 13 years (n = 2).
- Patients with pure vein of Galen malformations (n = 1), dural AVMs (n = 3), direct pial fistula (n = 3) or any other (n = 2) type of intracranial vascular malformation occasionally confused with a brain AVM [21].
- Patients with incomplete hospital record (n = 5).

## Operational definitions

### Brain AVM (BAVM)

We defined a brain AVM as an anastomosis of non-nutritive blood vessels in the brain parenchyma, in which arteriovenous shunting occurred in a central nidus (the tangle of vessels in which usually multiple arteries and veins converge). Diagnoses were deemed to be definite if there had been unequivocal evidence of a characteristic serpiginous cluster of calcified or enhancing vessels on computed tomography, flow voids on magnetic resonance imaging, or arteriovenous shunting on catheter angiography.

### Venous ectasia

Venous ectasia/varix is defined as at least a doubling of the diameter of the draining vein.

### Intra nidal aneurysm

An aneurysm arising in or near the nidus of an AVM.

### Venous outlet stenosis

Abnormal narrowing of a vein draining an AVM nidus.

### Transit time and flow

Flow pattern through the nidus was calculated by transit time seen in DSA. The transit time through the AVM is defined as the time interval between initial visualization of injected contrast entering the arterial segment and its subsequent passage into the venous drainage threshold. The classification of slow to medium and high flow was done arbitrarily by the observer experience. But all the slow to high flow AVM had less than normal transit time.

### Feeding artery

A feeding artery was defined as any intracranial vessel that angiographically contributed arterial flow to the malformation. The AVM nidus was defined as the vascular mass included in the AVM size measurement. Intranidal aneurysms were coded when visualized early after angiographic injection, e.g., before substantial venous filling had occurred. Infundibula, arterial ectasias (i.e., dilated feeding vessels), and intranidal aneurismal dilatations seen during the venous angiographic phase only were not coded as arterial aneurysms.

### Angiopathic AVM

It was defined as a presumed diagnosis for a peculiar type of large brain arteriovenous malformations (AVMs) that demonstrated distinctive angiogenetic features by which they could be separated from “classical” brain AVMs. This denomination was strictly based on angiographic evidence of nonfocal angiogenetic activity, i.e., the presence of transdural supply and stenoses of feeding arteries. Other distinctive features were the absence of dominant feeders to a large nidus (often lobar or even hemispheric), the small size of the draining veins in relation to the size of the arteriovenous shunting zone, the presence of intermingled brain between the vascular spaces as demonstrated by MRI, and the above mentioned transdural supply and presence of proximal arterial stenoses.

### Treatment, outcome and follow up

Patients were treated either by endovascular embolization or by microsurgical resection or kept in conservative management. Factors that guided the selection of method of treatment included patient characteristics (e.g., age, co-morbidity), location of the AVM, the cost involved and also desire of the patient. Each case was discussed with the Department of Neurosurgery before applying any intervention. Appropriate cases were referred to Department of Neurosurgery for microsurgical excision. Some patients could not afford any therapy or had high risk of any operative procedure was treated conservatively. They were kept on medication to control the co-morbid conditions e.g., management of hypertension, diabetes etc.

The primary outcome was all cause of case fatality during treatment and before discharge from hospital. The secondary outcome was intracranial hemorrhage beyond 30 days of treatment (hemorrhage within 30 days was categorized as a complication of treatment) along with other complications (e.g., post operative seizure). Follow up was done at and beyond third month and outcome was evaluated with Modified Rankin Scale (MRS) [22] score before and after intervention.

This study was approved by the ethical review committee of Dhaka Medical College Hospital.

## Results

### Demographic features

The mean age at diagnosis was 30.3 years with a standard deviation of ±14.3. Majority were teenagers (30 %) with an interquartile range (Q3–Q1) of 22 years.

### Clinical characteristics

Intracranial hemorrhage was the commonest (70 %) type of presentation at diagnosis.

Headache (50 %), altered consciousness (50 %), vomiting (40 %) and seizure (40 %) were also frequently reported by the patients. A few also experienced vertigo (25 %), loss of balance (15 %), disturbance of speech and vision (10 %) (Table 1).

**Table 1:** Clinical features at presentation of AVM patients

Clinical features	Number	Percentage
Intracranial hemorrhage	42	70
Headache	30	50
Altered consciousness	30	50
Vomiting	24	40
Seizure	24	40
Vertigo	15	25
Loss of balance	9	15
Visual disturbance	6	10
Disturbance of speech	6	10

### Morphologic characteristics

Majority of the AVMs had feeders from anterior circulation (50 %), while only 20 % had feeders from posterior circulation alone. But 30 % of the AVMs received feeders from both the anterior and posterior circulation. Likewise 50 % of the AVM was supratentorial, 20 % was infratentorial and 30 % were both in supra and infratentorial location (Table 2). About 43 % had superficial location and 57 % had deeper location. Most of the AVMs (73.3 %) were supplied from the main feeders, whereas the rest from distal vessels (Table 3). Regarding venous drainage, AVMs drained mostly either to superficial (43.3 %) or deep (40 %) venous system. Only 16.6 % drained to both the venous systems (Table 4). The overall proportion of AVMs classified as small was 23.3 %, while 36.6 % of AVMs were medium in size, and 40 % were classified as large (Table 5). Most of the AVMs had slow to medium flow (60 %), while 30 % had high flow and 10 % had slow flow through the nidus (Table 6). An eloquent AVM location was found in 50 % of the patients. Intranidal aneurysm was found in 10 % AVM and angiopathic AVM in 13.3 % (Table 7).

**Table 2:** Location of the AVM in DSA

Location	Number	Percentage
Supratentorial	30	50
Infratentorial	12	20
Both	18	30
Superficial or cortical	26	43.3
Deep	34	56.7

**Table 3:** Identification of feeding vessels to AVM in DSA

Feeders	Number	Percentage
Anterior circulation	30	50
Posterior circulation	12	20
Both	18	30
Main vessels	44	73.3
Distal vessels	16	26.7

**Table 4:** Identification of venous drainage of AVM in DSA as evaluated by Spetzler–Martin Score

Venous drainage	Number	Percentage
Superficial	26	43.3
Deep	24	40
Both	10	16.6

**Table 5:** Nidus size of cerebral AVM as evaluated by Spetzler–Martin Score after DSA

Venous drainage	Number	Percentage
S_1_: <3 cm	14	23.3
S_2_: 3–6 cm	22	36.6
S_3_: >6 cm	24	40

**Table 6:** Pattern of flow through the AVMs

Flow	Number	Percentage
Slow	6	10
Medium	36	60
High	18	30

**Table 7:** DSA findings of the study patients (n = 60)

DSA findings	n	%
Positive for eloquence	30	50
Intranidal aneurysm	6	10.0
Angiopathic AVM	8	13.3

**Table 8:** Treatment, follow-up and outcome (n = 60)

Treatment modality	Outcome						
	Before discharge			Follow up			
	(MRS: 6)	(MRS: 3–5)	(MRS: 0–2)	(MRS: 6)	(MRS: 3–5)	(MRS: 0–2)	Rebleed
Endovascular	1	2	28	0	0	30	2
Surgical approach	1	2	8	0	1	8	3
Conservative	3	4	11	5	1	3	9

### Treatment and follow up

The method of treatment applied along the short term outcome is listed in Table 8. Out of 60 patients 18 patients were treated conservatively and the rest underwent either endovascular embolization (31) or surgical excision (11) of the AVM. Among the patients undergoing endovascular procedure complete occlusion of the AVM nidus was achieved in 12 patients and partial occlusion in 19 patients. More than one session of embolization was required in six cases. Two patients developed transient neurologic deficit in the form of hemiparesis (MRS-3) and one patient (3.2 %) died (MRS-6) about 4 h after the procedure from rebleed. Rest of the patients had MRS of 0–2 at discharge. Among those treated surgically, one died per operatively due to rupture of intranidal aneurysms (9.1 %). Two patients developed seizure post operatively and was controlled with antiepileptic medication before discharge. Three patient developed hemiparesis. On discharge out of the ten patients, eight had MRS 0–2 and two had MRS 4. Three patients (16.6 %) in conservatively treated group died before discharge. Among the rest 11 patients had MRS 0–2, four had MRS 3–5.

Patients were followed up for 1.3 ± 0.8 years with a range of 3 months to 2.5 years. Within the follow up period two patients (6.6 %) in endovascular group, three patients (30 %) in surgical group and nine patients (60 %) in conservatively treated group had rebleed. There were five deaths in the conservatively treated group; out of which four deaths were directly related to rebleed from BAVM and in one case the cause was unknown. But, there was no death in the intervention (surgical or endovascular) group. In conservatively treated group three patients had MRS 0–2 and one patient had MRS 4. Whereas only one patient in surgically treated group had permanent neurologic deficit (MRS-5) and the rest (eight patients) had MRS 0–2. In the endovascular group all patients (30) had MRS 0–2.

## Discussion

In this descriptive study we have tried to find out the clinical presentation, angiographic and radiologic morphology of BAVM along with the treatment and short term outcome of patients in Bangladesh. Patients most commonly presented with intracerebral hemorrhage, headache, altered consciousness and seizure. Neuroradiologic investigations showed they frequently had BAVM with large nidus and supplied by feeding vessels from anterior circulation and draining into superficial venous system. Patients treated either by endovascular embolization or microsurgical excision had better short term outcome than those managed conservatively. To our knowledge this is the first study evaluating clinical and morphological features of BAVM along with treatment outcome in Bangladesh.

Though brain AVMs can present at any age and equally in both sexes, like most of the published data we also have found that the mean age tends to be in the third decade of life [13, 23–27]. Similar to other published reports from all over the world, our patients most frequently presented with intracranial hemorrhage [13, 25–32]. The overall frequency of hemorrhage ranges from 35 to 81 % with an annual risk of 2–4 % in reported case series [29, 31, 33–45].

We found a good number of patients with headache and unconsciousness. But we could not differentiate from the hospital record, whether the reported headache in our patients were related to hemorrhage or not. We assume that the higher frequency of headache and altered consciousness might be related mostly to intracranial hemorrhage. But headache unrelated to hemorrhage had not been published in much of studies. Ghossoub et al. [46] in their study of 700 patients showed that unrelated headache may occur only in 6 % patients. The headaches are mostly non specific, unilateral (without any site preference) and female predominant. Seizure unrelated to hemorrhage was also not uncommon in our study and the frequency was consistent with reports published so far [47]. Mast et al. [47] reported seizure as an initial symptom can occur in 16–53 % patients. From a database of 198 patients in Columbia, the investigators found a seizure frequency of about 28 %, of which 49 % were generalized, 22 % focal and 22 % being focal with secondary generalized [48]. Some studies also have delineated the risk factors for seizure in AVM [48–50]. In another analysis of 1289 patients with AVM from three centers found 30 % generalised and 10 % focal seizure [51]. An earlier study of 102 patients found 45 % focal, 42 % generalised, 8 % psychomotor, and 7 % unspecific seizure presentations [52].

We found a relatively lower (50 %) proportion of patient with eloquent AVM location in comparison with other centers (69–74 %) [51]. In contrast to Hofmeister et al. [51], we found a higher frequency for superficial drainage of AVM which complies with the report of Stapf et al. [53]. The nidus size found in our study also differed from that of Hofmeister et al. [51] who also acknowledged a significant variation in nidus size in different centers. But Stapf et al. [53], showed a mean nidus size of 33 ± 17 mm in his study sample, who also observed a significant difference of nudus sizes across different age groups. The higher proportion of AVM feeder arteries from anterior circulation and from main vessels in our study may indirectly imply that majority of the AVMs had lobar and supratentorial location. This is also in line with the report from some other studies [53, 54]. Intranidal aneurysms are associated with AVMs in up to 10 % of patients in many series [12, 25, 54, 55] of studies like this one.

Treatment decisions for patients with (BAVMs) are based on natural-course and risk estimates weighed against outcome data from invasive intervention. Though neurosurgeons had been practicing microsurgery for BAVM in different institutions in Bangladesh for long, the endovascular method is relatively recent (since 2007) addition to modality of BAVM treatment. The overall mortality (MRS-6) and morbidity (MRS 3–5) of intervention (endovascular and surgical) was higher than those reported by Amor et al. [56] and Hamilton et al. [57]. This is probably due to the fact that these interventional treatments are relatively new in Bangladesh. But the death rate was five times higher in conservatively treated group which justifies the benefit of intervention. The outcome of treatment was also retained during follow up.

We had several limitations in this study. Firstly, the study sample was relatively low. Secondly, the retrospective nature did not allow us to further investigate any quarry that we had in our mind. Moreover, this cross-sectional study is merely observational and does not provide a longitudinal risk analysis. Finally, referral bias to specialized treatment centers may significantly influence demographic, clinical, and morphological characteristics of the local patient cohort. The possibility of a systematic error in the data analysis can therefore not be excluded.

## Conclusion

Intracerebral hemorrhages, headache, altered consciousness and seizure are common form of presentation of AVM at the time of diagnosis. Larger AVM nidus size at eloquent location, medium to slow flow with frequent feeders from main vessels of anterior circulation and draining to superficial venous systems are noticeable morphologic features in our study. Endovascular embolization or surgical excision of AVM are relatively safe and effective and provides better short term outcome than conservative approach.
